# Longitudinal changes in retinal microvasculature after panretinal photocoagulation in diabetic retinopathy using swept-source OCT angiography

**DOI:** 10.1038/s41598-020-80697-0

**Published:** 2021-01-08

**Authors:** Kiyoung Kim, Eung Suk Kim, Seung-Young Yu

**Affiliations:** Department of Ophthalmology, Kyung Hee University Medical Center, Kyung Hee University Hospital, 23, Kyungheedae-ro, Dongdaemun-gu, Seoul, Republic of Korea

**Keywords:** Retinal diseases, Diabetes

## Abstract

This study evaluated quantitative changes in microvascular parameters after panretinal photocoagulation (PRP) in diabetic retinopathy (DR), using swept-source OCT Angiography (SS-OCTA). A total of 27 treatment-naïve eyes were subjected to PRP and followed-up for > 12 months after the procedure. Foveal avascular zone (FAZ) area, macular perfusion density (PD), and vessel length density (VLD) were calculated on a 3 × 3 mm en face OCTA image and nonperfusion area (NPA) was obtained on a 12 × 12 mm en face OCTA image. One month after PRP, PD and VLD of superficial and deep capillary plexus decreased and subsequently, increased progressively across the next 12 months, with statistically significant differences (P = 0.015 and 0.02). Continuous decreasing trends in total NPA values was observed across 12 months after PRP (P = 0.125). A difference in PD of the superficial capillary plexus between baseline and 6 months post PRP, was significantly associated with the progression of DR, 12 months after PRP (OR 0.528; P = 0.025). We found significant longitudinal retinal microvascular changes after PRP in DR. Overall macular perfusion status was impaired and progressively recovered across the next 12 months, compared to the baseline. Additionally, the early treatment responses in PD can predict the long-term outcomes of PDR after PRP.

## Introduction

Diabetic retinopathy (DR) is the most common cause of vision loss in the working-age population globally^1^. Proliferative DR (PDR) is characterized by the formation of retinal neovascularization, potentially resulting in vitreous hemorrhage or tractional retinal detachment. Panretinal photocoagulation (PRP) is the gold standard for treatment of severe non-proliferative DR (NPDR) or PDR for preventing visual loss^2^. However, predicting individual outcome of PRP can be difficult, because some cases of PDR continue to progress, whereas others remain stable, after treatment with PRP. A proposed general mechanism of PRP involves absorption of the energy from the laser by the retinal pigment epithelium, leading to thermic destruction of oxygen-consuming photoreceptors, and thereby improving the oxygenation of the ischemic inner retinal layers^3^. The efficacy of PRP for PDR is related to the consequent reduced angiogenic drive and the recovery of blood flow autoregulation, which have a positive effect on the regression of neovascularization^4^.

Several human studies have reported changes in retinal and choroidal thickness after PRP^5,6^. The mechanisms responsible for the increase in macular thickness could be PRP-induced retinal vasodilation, inflammation, and edema^7^. However, previous studies have reported contradictory results on the subject of choroidal thickness, depending on the different inclusion criteria and varied durations of follow-up^6,8^. In addition, literature has reported that PRP influences the retinal blood flow, which has been studied using various imaging techniques, either in a single retinal blood vessel or the large retrobulbar vasculature^9,10^. These studies demonstrated large vessel constriction and an increased speed of blood flow, but an overall decrease in blood flow after successful PRP treatment^9,10^. While previous studies have explored the effects of PRP on large blood vessels, to the best of our knowledge, very few studies has addressed a detailed description of long-term microvascular changes.

Although fluorescein angiography (FA) remains the gold standard for the assessment of retinal perfusion and diagnosis of neovascularization in diabetic eyes, optical coherence tomography angiography (OCTA) has demonstrated superior ability in discriminating detailed morphological and vascular changes, especially near the fovea^11^. With the progression of DR, retinal capillary nonperfusion in OCTA is significantly and linearly correlated with disease severity. Also, distinct changes of capillary blood flow at two different level of capillary layers was reported^12^. However, one of the shortcomings of OCTA is the limited field of view. The introduction of swept-source OCTA (SS-OCTA) enabled wider scan size for the evaluation of retinal diseases, which cause significant alterations beyond the posterior pole^13^.

The main purpose of the present study was to evaluate the effect of PRP on the vascular perfusion status by means of wide-field SS-OCTA, and to investigate whether the microvascular parameters could be used as non-invasive markers in the prognosis of DR.

## Results

In total, 27 eligible eyes of 27 patients were examined in this study. The baseline characteristics of the study subjects are summarized in Table 1. Twelve months after PRP, six eyes (22.2%) out of 27 demonstrated progression of DR according to the FA examination. Baseline DR grade for five of these eyes were PDR and one case was severe NPDR.

**Table 1:** Comparison of baseline clinical characteristics and foveal microcirculation parameters in patients with type 2 diabetes.

Eyes (n)	27
**Grade of DR (n)**	
Severe NPDR	12
PDR	15
Age (years)	55 ± 7.9 (44–67)
Gender (male:female)	12:15
Hypertension (yes:no)	11:16
Diabetic duration (years)	8.14 ± 6.82 (3–20)
HbA1c (%)	7.53 ± 1.20 (6.2–10)
Visual acuity (logMAR)	0.12 ± 0.14 (0–0.5)
Central foveal thickness (µm)	268.9 ± 30.3 (212–327)
mGCIPL thickness (µm)	83.71 ± 12.63 (62–101)
**FAZ area (mm**^**2**^**)**	
SCP layer, 3 × 3 mm	0.39 ± 0.15 (0.13–0.63)
DCP layer, 3 × 3 mm	0.48 ± 0.13 (0.25–0.66)
**Perfusion density (%)**	
SCP layer, 3 × 3 mm	32.70 ± 8.63 (23.9–43.2)
DCP layer, 3 × 3 mm	32.71 ± 7.55 (20.5–52.7)
**Vessel length density (mm**^**−1**^**)**	
SCP layer, 3 × 3 mm	10.27 ± 1.99 (7.7–13.8)
DCP layer, 3 × 3 mm	10.56 ± 2.78 (5.9–15.5)
Nonperfusion area, 12 × 12 mm (mm^2^)	5.60 ± 3.93 (1.1–14.5)

*DCP* deep capillary plexus, *FAZ* foveal avascular zone, *HbA1c* glycosylated hemoglobin, *mGCIPL* macular ganglion cell/inner plexiform layer, *NPDR* non-proliferative diabetic retinopathy, *PDR* proliferative diabetic retinopathy, *SCP* superficial capillary plexus.

### Spectral domain-OCT (SD-OCT) parameter

**Figure 1:**
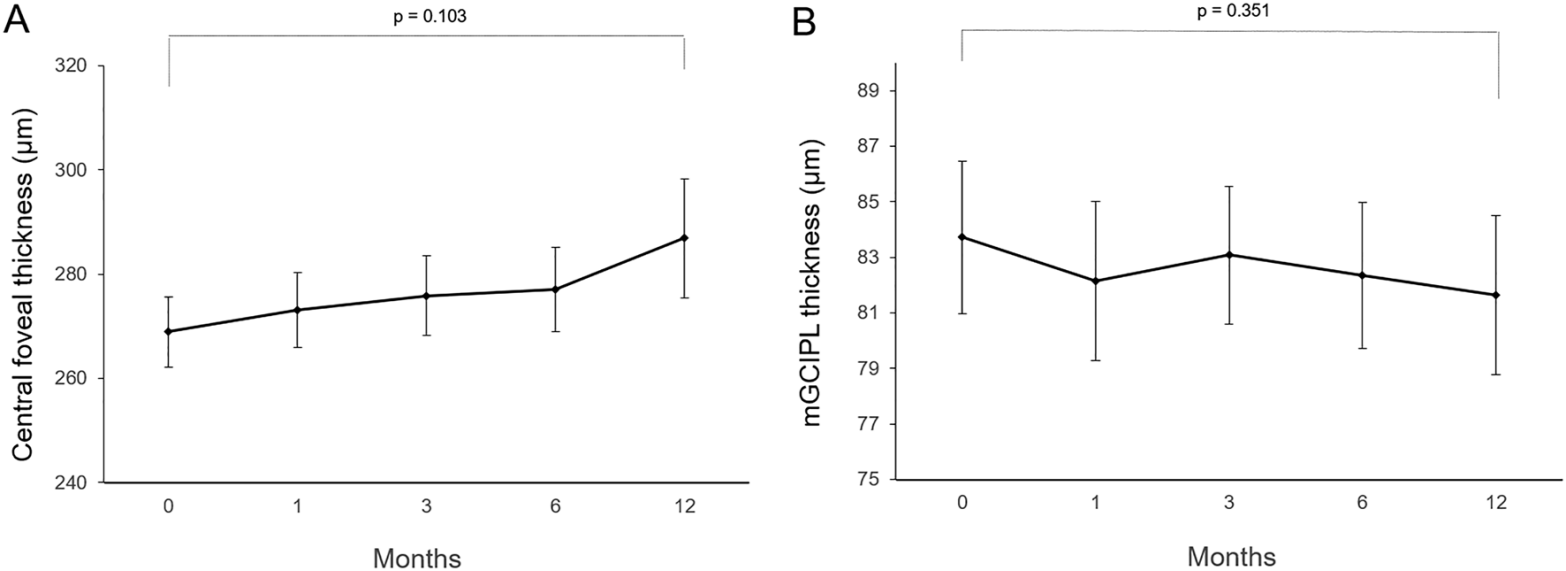
Mean longitudinal changes in (**A**) central foveal thickness, (**B**) macular ganglion cell-inner plexiform layer (mGCIPL) thickness before and 12 months after PRP.

The mean central foveal thickness (CFT) of the 27 eyes at the baseline was 268.9 μm and at 1, 3, 6, and 12 months post PRP were 273.1 μm, 275.9 μm, 277.1 μm, and 286.9 μm, respectively (Fig. 1A). A continuous increasing trend was observed in CFT across 12 months, compared to the baseline measurements (P = 0.103). On follow-up examinations, the mean macular ganglion cell-inner plexiform layer (mGCIPL) thickness was observed to decrease at 1 (82.14 μm), 3 (83.07 μm), 6 (82.35 μm), and 12 months (81.64 μm) after PRP, compared to the baseline (83.71 μm) values (Fig. 1B). Following repeated-measure analysis of variance (RM-ANOVA) indicated no statistically significant difference between the measurements obtained on post-PRP follow-up examinations (P = 0.351). Mean subfoveal choroidal thickness (SFCT) was 275.7 ± 66.9 mm at baseline, 272.8 ± 62.0 mm at 1 month, 270.8 ± 66.2 mm at 3 months, 265.4 ± 69.0 mm at 6 months, 260.2 ± 61.1 mm at 12 months post-PRP. Continuous decreasing trend was observed in SFCT across 12 months without statistically significance (P = 0.108).

### OCTA parameters

**Figure 2:**
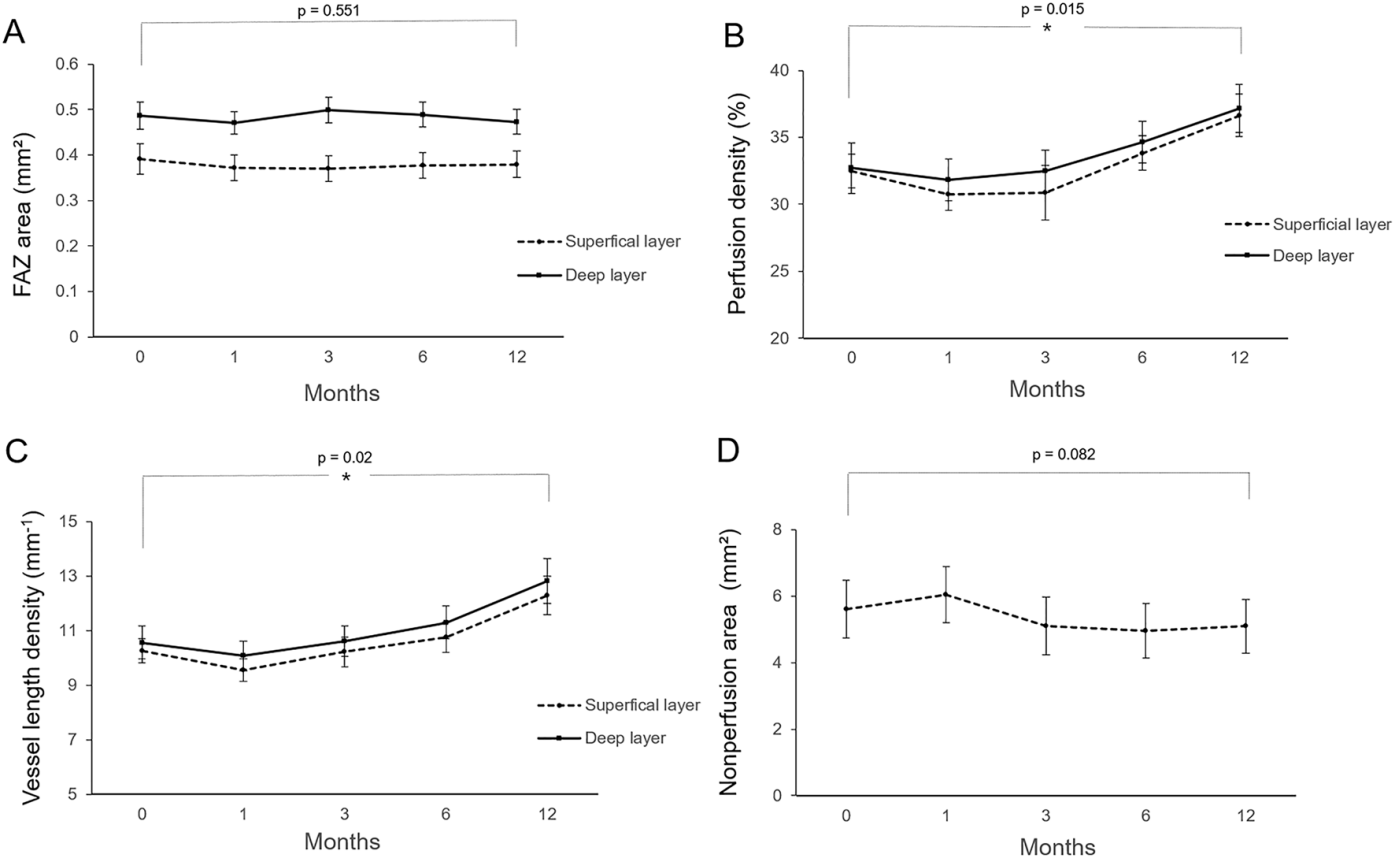
Mean longitudinal changes of microvascular parameters from optical coherence tomography angiography (OCTA) before and 12 months after PRP. (**A**) Foveal avascular zone area, (**B**) macular perfusion density, (**C**) macular vessel length density, and (**D**) total nonperfusion area.

Results of longitudinal changes in the microvascular OCTA-parameters are shown in Fig. 2. The baseline values of foveal avascular zone (FAZ) area in superficial capillary plexus (SCP) and deep capillary plexus (DCP) were 0.39 mm^2^ and 0.48 mm^2^, respectively, and were observed to be slightly decreased after 12 months, to 0.38 mm^2^ and 0.47 mm^2^, respectively. The difference was not observed to be statistically significant on RM-ANOVA, across the 12-month follow-up points (P = 0.551).

After PRP treatment, perfusion density (PD) and vessel length density (VLD) of the SCP showed a slight decrease after 1 month (31.82%, 9.55 mm^−1^) compared to the baseline measurements (32.7%, 10.27 mm^−1^). However, on examinations at 3 and 12 month follow-ups, the values were observed to increase continuously, beyond the baseline values, with measurements of 34.63%; 10.76 mm^−1^ at 6 months and 37.16%; 12.29 mm^−1^ at 12 months. The difference between baseline and 12 months was found to be statistically significant (P = 0.015). Longitudinal changes observed in the PD and VLD of DCP showed a similar pattern. The lowest values of both PD and VLD of DCP were observed on the 1 month follow-up examination, and the values progressively increased across the next 12 months, with a statistically significant difference between the measured values (P = 0.02).

The total nonperfusin area (NPA) on 12 × 12 mm total retinal en face OCTA image was observed to be increased on the 1 month (6.04 mm^2^) follow-up, and then progressively decreased across the next 12 months (5.09 mm^2^). RM-ANOVA showed no statistically significant difference, when compared to baseline values (5.60 mm^2^, P = 0.082).

### Risk factors for the progression of DR after PRP

Table 2 shows the results of logistic regression analysis in terms of baseline OCT and OCTA metrics to DR progression, 12 months after PRP. In the univariate analysis, lower increase in PD of SCP at 6 months after PRP was significantly associated with the progression of DR at 12 months. (OR 0.468; P = 0.031). In the multivariate analysis, these associations remained significant after adjustments for age, sex, duration of diabetes, HbA1c level, mean arterial blood pressure (MABP) and severity of DR at the baseline (OR 0.528; P = 0.025). Other OCTA metrics did not show significant associations with the progression of DR after PRP treatment.

**Table 2:** Logistic regression analysis of Optical Coherence Tomography Angiography Metrics and Risk of Diabetic Retinopathy Progression, 12 months after PRP.

OCT metrics	Univariate		Multivariate	
	OR (95% CI)	P value	OR (95% CI)	P value
Central foveal thickness	1.020 (0.986–1.055)	0.250	1.042 (0.991–1.095)	0.352
mGCIPL thickness	1.093 (0.985–1.214)	0.093	1.056 (0.944–1.168)	0.118
**OCTA metrics**				
FAZ area (SCP)	0.005 (0.001–8.804)	0.165	0.009 (0.003–11.84)	0.122
FAZ area (DCP)	0.109 (0.001–141.2)	0.544	0.144 (0.062–90.46)	0.351
Perfusion density (SCP)	0.760 (0.447–1.293)	0.312	0.788 (0.468–1.335)	0.258
Perfusion density (DCP)	0.900 (0.629–1.288)	0.565	0.892 (0.629–1.205)	0.467
Vessel length density (SCP)	0.907 (0.747–1.100)	0.321	0.882 (0.725–1.092)	0.322
Vessel length density (DCP)	0.967 (0.862–1.085)	0.569	0.982 (0.823–1.068)	0.488
Nonperfusion area	0.941 (0.726–1.220)	0.647	0.955 (0.688–1.252)	0.598
**Difference of OCTA metrics (6 months–baseline)**				
FAZ area (SCP)	4.713 (0.321–77.84)	0.773	2.502 (0.119–18.55)	0.555
FAZ area (DCP)	0.004 (0.001–43.22)	0.187	0.013 (0.009–9.551)	0.180
Perfusion density (SCP)	0.468 (0.234–0.937)	0.031*	0.528 (0.286–0.983)	0.025*
Perfusion density (DCP)	0.589 (0.647–1.015)	0.062	0.555 (0.611–1.003)	0.055
Vessel length density (SCP)	0.827 (0.339–1.026)	0.069	0.791 (0.301–1.012)	0.065
Vessel length density (DCP)	0.868 (0.732–1.029)	0.102	0.852 (0.708–1.012)	0.155
Nonperfusion area	1.163 (0.624–2.167)	0.635	1.295 (0.665–2.202)	0.505

*CI* confidence interval, *DCP* deep capillary plexus, *FAZ* foveal avascular zone, *mGCIPL* macular ganglion cell/inner plexiform layer, *SCP* superficial capillary plexus.

*Multivariate model adjusted for age, sex, and baseline DR severity.

## Discussion

The pathophysiology of early DR, prior to the development of significant vasculopathy, has not been clearly defined. Several studies indicate that DR progression is accompanied by significant retinal microvascular changes and neurodegeneration^14,15^. However, the correlation between these pathologies and their contribution to clinically visible retinopathy is still unclear. In the early stages of DR, hypoperfusion may contribute to pro-inflammatory changes such as leucocytic adherence to the retinal capillaries and vaso-occlusion; resulting in capillary dropout and the development of a progressive, irreversible ischemic hypoxia^16^. Although FA remains the gold standard for the visualization of the retinal neovascularization in PDR, the mechanism of large vessel hemodynamic alteration affecting macular microvascular distribution, has not been established yet. Recent advances, like higher-axial-resolution OCTA, have the potential to generate superior axial resolution and the capability to segment the microvascular network from different retinal layers. The OCTA system has demonstrated its superiority in quantification of microvascular density, capillary dropout, and FAZ area in diabetic patients^17^. Moreover, several studies have reported the OCTA parameters of macular perfusion state in the different stages of DR, with or without diabetic macular edema (DME)^18,19^.

In argon laser photocoagulation, the laser energy is absorbed by the retinal pigment epithelium (RPE) and generates thermal energy to the outer retina. Heat damage in the inner retinal layers causes edema due to increased vascular permeability and presents as increased retinal thickness^20^. As expected, the authors observed a mild increase in macular thickness during the 12 months post PRP. Previous studies have demonstrated large vessel constriction and overall blood flow decrease, following the treatment of PDR with PRP, by means of a host of techniques^4,9,10^. Color Doppler imaging, laser interferometry, and laser Doppler flowmetry have been used to obtain quantitative data on retinal and choroidal circulation in eyes with DR, before and after PRP^21^. In addition, laser speckle flowgraphy has been used to measure the reduced retinal blood flow, during and 6 months after the PRP^22^. However, previous reports on choroidal blood flow at the macula area, following PRP treatments, have been contradictory^21,23^.

**Figure 3:**
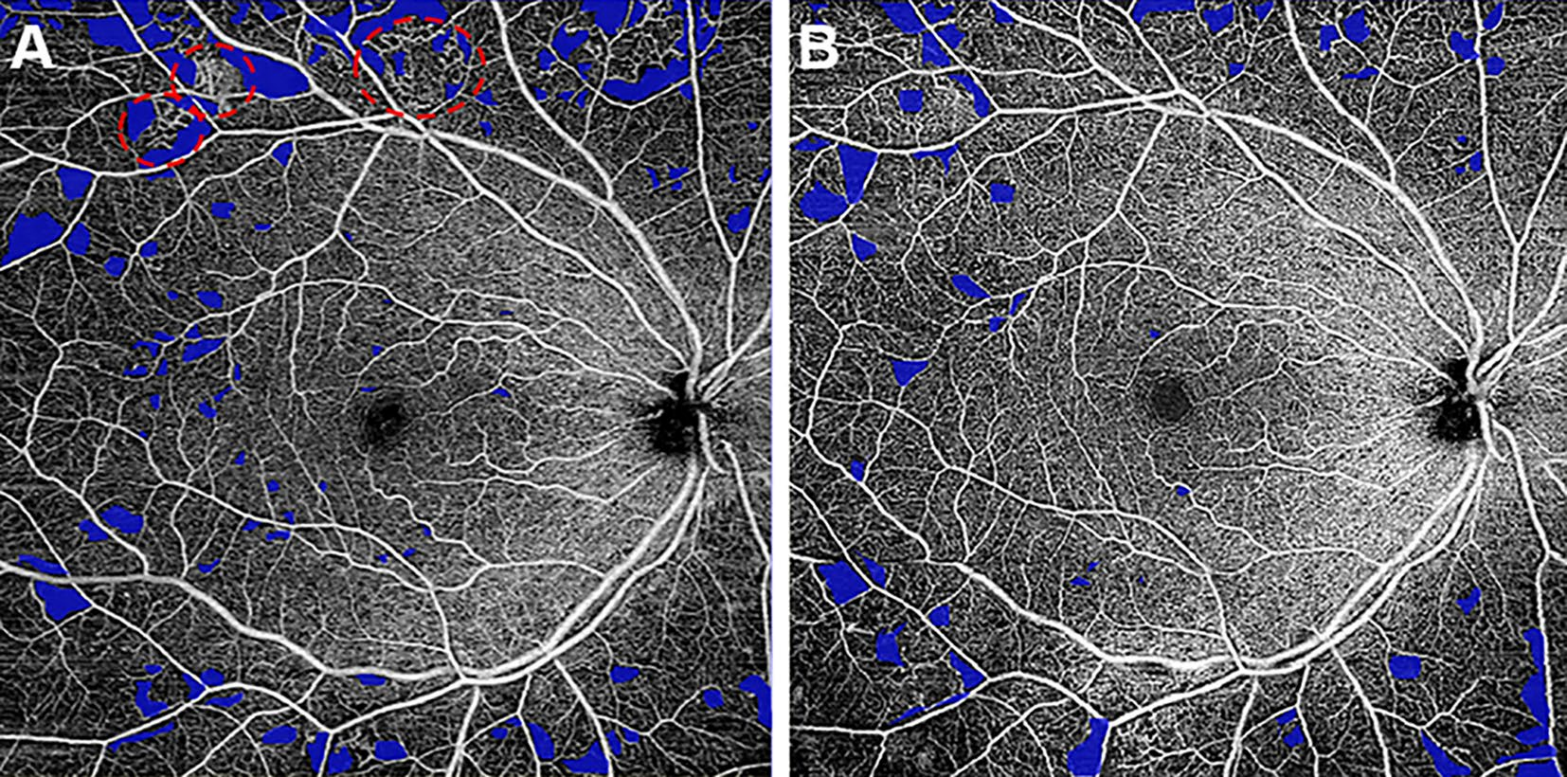
(**A**) Representative SS-OCTA images for PDR cases that had mid-peripheral neovascularization and IRMAs (red circles). (**B**) Three months after PRP treatment, neovascularization and IRMAs disappeared and re-established with retinal capillaries. Overall nonperfusion area (blue color coded) reduced and capillary perfusion density and vessel length density improved after PRP.

Recent advances like OCTA permit the quantification of macular capillary modulations across the superficial and deep layers, in response to PRP. This finding has not been reported by previous studies, which were limited to the study of large vessel hemodynamics. Therefore, we performed serial follow-up OCTA imaging to compare with baseline measurement by 12 months post PRP. Fawzi et al.^24^ reported an overall increase in the OCTA flow metrics of all capillary layers in the macula, 6 months after PRP. Mirshahi et al.^25^ reported that foveal vascular density, measured using OCTA, increased 3 months after PRP. In the current study, decrease of PD and VLD, 1 month after PRP implies the effect of acute inflammation of the retinal tissue, subsequent to laser treatment. Consequently, we observed an overall increase in PD and VLD at both capillary plexuses, 12 months after PRP and there was a significant difference, when compared to the measurements taken 1 months after PRP. Possible underlying mechanism for the improved flow in the remaining macular capillaries could be re-establishment of macular microvasculature, from regression of peripheral neovascularization or intraretinal microvascular abnormalities (IRMA). A representative case of PDR treated with PRP was shown in Fig. 3A. As mid-peripheral neovascularization and IRMA disappeared, capillary perfusion improved and NPA decreased after 6 months (Fig. 3B). Conversely, our analyses did not reveal any significant alteration in the FAZ area following PRP, which was consistent with previous literature^25,26^. Lorusso et al.^26^ in a similar study, investigated the alteration of OCTA parameters following PRP. Contradictory to our results, they did not observe any changes in the PD and FAZ area. This discrepancy can be due to the different methods employed to measure OCTA parameters and the diverse follow-up periods. Primarily, we focused on the macular microvascular status, without considering the well-known vasoconstrictive effect on larger vessels, following PRP treatment. By excluding the major branches of retinal vessels on OCTA, we calculated the capillary density selectively as the percentage of vascular voxels on en face projection angiograms. Furthermore, the duration of follow-up in the current study extended up to 12 months after PRP, compared to previous studies with relatively shorter duration of follow-ups, such as one to 3 months after PRP. In the present study, the measured values of PD and VLD increased continuously, across the 12-month follow-up period, and the difference was found to be statistically significant.

The exact mechanism of the early decrease in PD and VLD, following PRP, is still unknown. One possible explanation for this reduction is that the capillary flow following PRP might be associated with early PRP-induced inflammation. After sufficient time, the inflammation probably subsides and subsequently, the resumption of the autoregulatory functions of retinal vasculature occurs. PRP is known to cause the upregulation of pro-inflammatory cytokines, such as intercellular adhesion molecule-1 (ICAM-1) and monocyte chemotactic protein-1 (MCP-1), vascular endothelial growth factor (VEGF) which in turn stimulates vascular permeability and capillary dropout^27^. Hence, it can be naturally hypothesized that these changes negatively add up to the pre-existing condition of DR associated maculopathy, further reducing the perfusion of the posterior pole, increasing the macular ischemia and enlarging the FAZ area. Qualitative and quantitative abnormalities such as reduced perfusion within the posterior pole and increase of the macular ischemia have been described in subjects with uveitis, in recent OCTA studies, which demonstrated a significantly lower parafoveal capillary density in the SCP layer of eyes with uveitis, when compared to healthy eyes^28^. Abnormalities in retinal vasculitis included capillary dropout or loss of the SCP layer^29^. Flow indices in perifoveal area were decreased with OCTA, suggestive of ischemia; this correlated with a greater severity of Birdshot chorioretinopathy^30^.

A recent study reported the longitudinal outcomes regarding the changes in NPA, following anti-VEGF treatment in DR. Reperfusion of vessels or capillary network was not detected in NPA using two imaging techniques (FA and SS-OCTA), following three anti-VEGF injections, in eyes with DR^31^. Similarly, in this study, PRP did not significantly change NPA in 12 × 12 mm field of SS-OCTA, across the 12 months after PRP.

The optimal laser protocol for PRP and the recommended end point for laser treatment have not been determined yet, because the reaction to PRP varies from patient to patient. Some earlier studies have tried to determine the prognostic factors, which influence the clinical outcomes of PRP. Grunwald et al. suggested using the restored response to the hyperoxic challenge as a tool to determine the success of PRP^24^. Hammer et al.^32^ demonstrated a trend towards lower retinal venular oxygen saturation in PDR patients who underwent PRP, compared to treatment-naïve PDR. They also suggested that retinal oxygen saturation could act as an early indicator of PRP treatment response and can be a valuable tool for the individualized treatment^33^. Furthermore, it was found that increasing retinal vascular arteriolar oxygen saturation, following PRP, trended towards a lower risk of active PDR^34^. Earlier studies have found a decrease in fractal dimension in patients with PDR, who underwent PRP^35^. Torp TL et al.^36^ found an increase in the arteriolar vessel caliber, which could represent a positive response to the PRP treatment, with a lower hypoxic load on the retina and a subsequent autoregulatory arteriolar dilation. The present study demonstrates that an increase in the parafoveal PD 6 months after PRP, can independently predict the progression of PDR, and might be a potential non-invasive OCTA marker of DR activity.

A major limitation of this study is the relatively small sample size, which may not be sufficient to find a definite temporal relationship between PRP and microvascular changes. Moreover, the current study used 3 × 3 mm scans for the calculation of PD and VLD, which were not wide enough to include the vascular arcades or the entire posterior pole. Rabiolo A et al.^37^ reported that interrater reliability for the quantified vessel density was relatively poorer in 6 × 6 mm, 12 × 12 mm than 3 × 3 mm scan in DR. The decrease of image resolution to larger angiocubes could have been strongly affected by the visualization of small capillaries or blocking artifacts, especially when a threshold is applied. Future research, which employs SS-OCTA devices with higher scan speed, density, with wider range of scans might provide more reliable information on the subject. Finally, the effect of PRP can vary considerably, depending on undetermined factors including spot size, number and the extent of PRP. Therefore, all the procedures were performed by a single operator, in order to minimize the effect of confounding factors in the study.

In summary, the current study observed that PRP treated eyes showed significant changes of macular capillaries, with increased PD and VLD at both capillary layers, suggesting an effective perfusion of the posterior pole. We assume that redistribution of blood flow from the periphery to the macular region results in reorganization of capillary networks. In view of the fact that the postoperative changes of macular PD closely reflect the disease activity, an individualized treatment approach might be available. This might be helpful in determining the necessity of additional PRP or anti-VEGF injections, in the early phase after PRP, to prevent the progression of DR.

## Methods

### Subjects

The subjects were retrospectively recruited patients with type 2 diabetes who received PRP treatment in the Department of Ophthalmology, Kyung Hee University Hospital, between October 2017 and April 2018. Institutional review board of Kyung Hee University Hospital approved the study protocol, and informed consent was obtained from each participant. This study was conducted in accordance with the tenets of the Declaration of Helsinki. In accordance with the recommendations of the Early Treatment Diabetic Retinopathy Study (ETDRS) Research Group, PRP was performed in patients with severe NPDR or proliferative DR. Exclusion criteria were as follows: patients with history of any intravitreal injection treatment or laser photocoagulation; patients having myopia with > − 5.0 diopters and intraocular pressure (IOP) of > 21 mmHg; and patients with retinal disease other than DR such as age-related macular degeneration and retinal vein occlusion. In addition, eyes with vitreous hemorrhage or significant DME, which deteriorated OCTA signal were also excluded, after an initial examination.

All subjects underwent a complete ophthalmic examination, including best-corrected visual acuity (BCVA), slit lamp biomicroscopy, detailed fundus examination, SD-OCT, SS-OCTA, and FA before the commencement of PRP. Follow-up examinations were performed at 1, 3, 6 and 12 months after PRP. FA was performed 12 months after PRP, in order to evaluate the progression of DR following PRP. Progression of PDR was defined as the development of retinal neovascularization, increased area of neovascularization, or increased area of fluorescein leakage on FA.

### Panretinal photocoagulation

Panretinal photocoagulation was performed as an outpatient procedure. The pattern scan laser photocoagulation system uses a frequency-doubled 532 nm wavelength neodymium: yttrium aluminum garnet laser (PASCAL^Ⓡ^, Topcon Medical Laser Systems). Photocoagulation was performed, by a single retinal specialist (E.S.K), in three sessions, with intervals of 1 week between each session. In accordance with the ETDRS protocol, each eye was subjected to 1200–1600 burns. In order to create yellowish white, coagulative spots, duration and power were adjusted to 0.02 s and 200–500 mW, respectively.

### Macular thickness measurement

Macular cube (512 × 128 scan) was obtained with Cirrus HD-OCT (Carl Zeiss Meditec, Inc., Dublin), in order to measure CFT. The built-in algorithms software can automatically identify the outer boundary of the macular retinal nerve fiber layer (RNFL) and inner plexiform layer (IPL). The difference between the RNFL and the IPL outer boundary segmentation yields the mGCIPL thickness. The average mGCIPL thickness of six sectors was used for statistical analysis.

### Swept-source OCTA imaging

Using SS-OCTA system (PLEX^®^ Elite 9000, Carl Zeiss Meditec, Dublin, CA, USA), both 3 × 3 and 12 × 12 mm raster imaging were performed and each scan pattern consisted of 500 A-scans per B-scan, at 500 B-scan positions. Slabs of SCP and DCP were automated and segmented by the built-in software. A minimum signal strength threshold of 7 of 10 was required for inclusion, and significant motion artifact or incorrect segmentation, were excluded and repeated. A series of quantitative OCTA metrics of SCP and DCP, including FAZ area, PD, VLD, and NPA were measured. PD was defined as the percentage of the total area occupied by blood vessels within the 3 × 3 mm, and VLD as the ratio of the total skeleton area over the 3 × 3 mm area.

**Figure 4:**
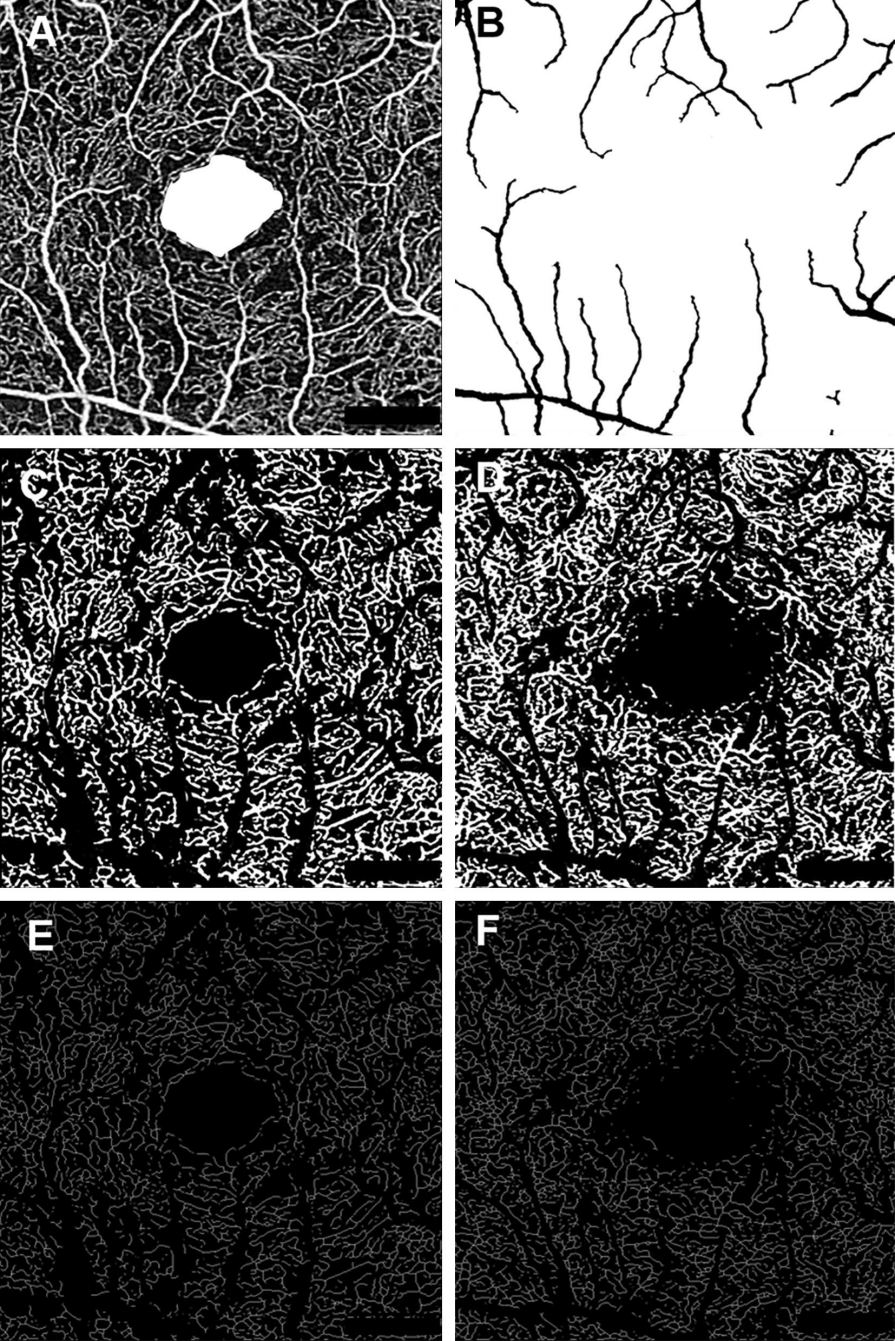
Imaging process for the quantification of microvascular parameters from swept- source OCTA images. (**A**) Superficial capillary plexus of 3 × 3 mm angio en face images. The foveal avascular zone area was manually outlined. (**B**) Separated large vessels image is subtracted from original OCTA images. (**C**,**D**) Binary images used to calculate perfusion density of superficial and deep capillary plexus layer. (**E**,**F**) Skeletonized images of corresponding binary images used to calculate vessel length density of superficial and deep capillary plexus layer.

**Figure 5:**
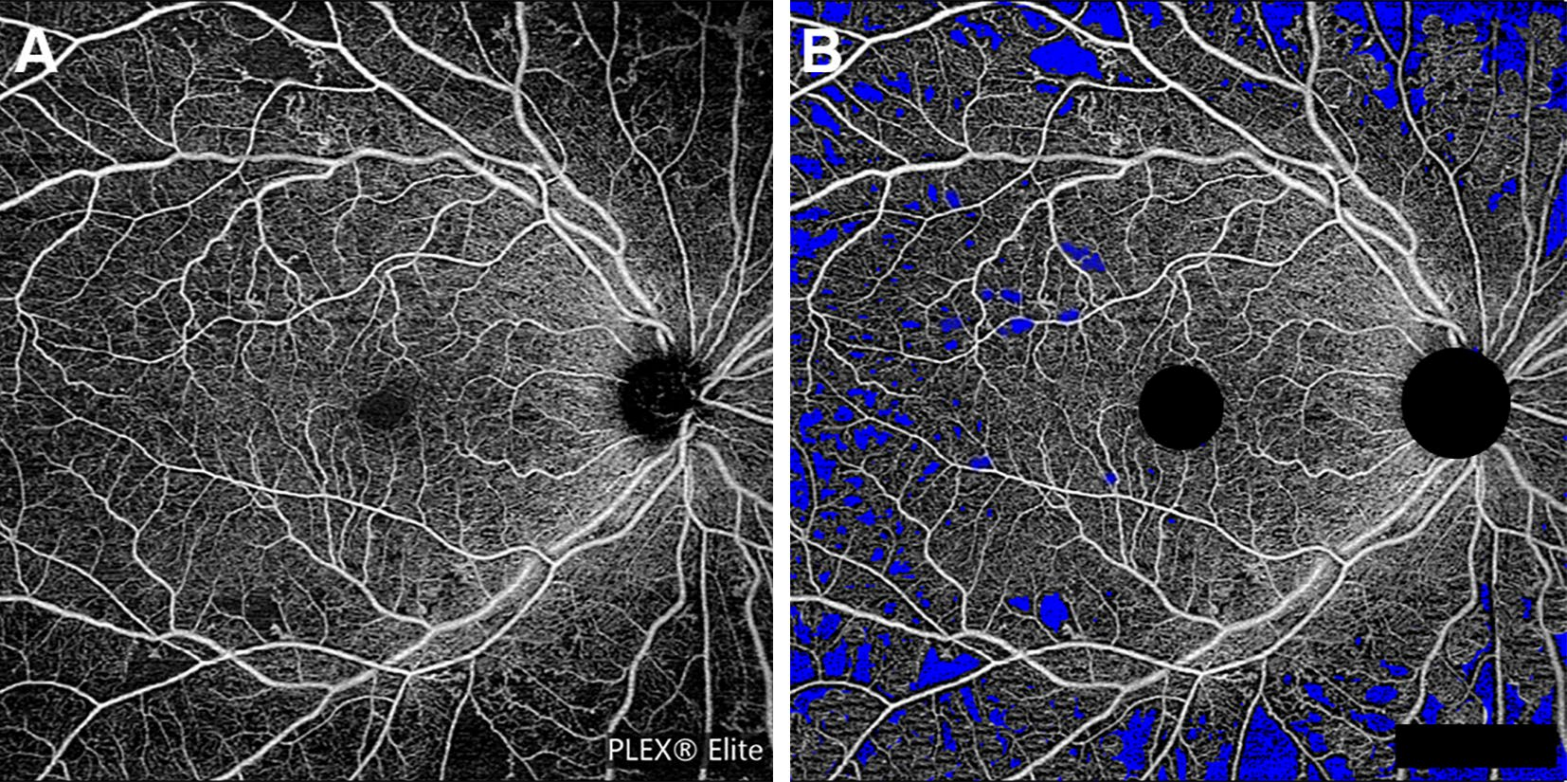
Representative images for the calculation of nonperfusion area. (**A**) Superficial capillary plexus of en face OCTA image of 12 × 12 area. (**B**) Nonperfusion area was detected and filled with blue color by semi-automatic threshold algorithm.

In order to quantify microvascular structure, first, the image encompassing large vessels was separated from the 3 × 3 mm image of SCP layer. Automated algorithm was applied considering thickness and intensity of large vessel. We empirically defined diameter of large vessel as 7 × 7 pixel on enface image. Then, we separated the major vessels on high intensity over 0.80 considering decorrelation signal of OCTA. Vessel threshold was defined by a multiple of maximum intensity of pixel and constant range from 0.65 to 0.90, according to the consensus of two physicians. Median filter was applied, in order to exclude small vessels and high intensity signal noise. Finally, large vessels were masked out to generate the capillary image. The perfusion density was calculated on binarized images, using an Image J (National Institutes of Health [NIH], Bethesda, MD, USA) ‘mean threshold’ algorithm. Binary images were then converted into skeletonized images, where vessels were reduced to a width of one pixel. Skeletonized images were used to calculate VLD using Image J program. Figure 4 represents imaging process of en face binarized and skeletonized images from the SCP and DCP layers. Secondly, NPA was measured based on a total retinal slab of 12 × 12 mm SS-OCTA image and semi-automatically quantified using MATLAB software (R2013b, MathWorks, Inc., Natick, MA, USA). In the context of the current study, NPA was defined as a contiguous region without microvasculature, composed of rectangular 17 × 17 (289) pixels (≥ 0.04 mm^2^). Area threshold was defined as 250 pixels. The threshold value to binarize the NPA was based on the method previously described by Kim et al.^28^ (250 pixels on the same SS-OCTA instrument). Fovea and optic disc area were manually excluded and finally NPA was calculated (Fig. 5).

### Statistical analyses

Statistical analyses were performed using SPSS software (version 18.0; SPSS, Chicago, IL, USA). Microvascular parameters after 1, 3, 6, and 12 months PRP were compared to the baseline values, using RM-ANOVA and the Bonferroni post hoc test. Correlations were assessed using the Pearson correlation or Spearman rank correlation test. Multiple logistic regression analysis was performed to find significant predictors for the progression of DR with adjustments for sex, age, duration of diabetes, HbA1c, mean arterial blood pressure.
